# *GATA3* germline variants in childhood pre-B acute lymphoblastic leukemia: association with *CRLF2* overexpression and overweight in Mexican patients

**DOI:** 10.3389/fonc.2025.1533756

**Published:** 2025-05-12

**Authors:** Adriana Reyes-León, Eduardo Castro-Vargas, Ma. del Rocío Juárez-Velázquez, Daniel Martínez Anaya, Consuelo Salas-Labadía, Dafné Moreno-Lorenzana, César Alejandro Galván-Díaz, Norma López-Santiago, Eduardo García-Padilla, Alda Daniela García-Guzmán, Isabel Medina-Vera, Patricia Pérez-Vera

**Affiliations:** ^1^ Laboratorio de Genética y Cáncer, Instituto Nacional de Pediatría, Ciudad de México, Mexico; ^2^ Becario de la Dirección General de Calidad y Educación en Salud, Secretaría de Salud, México, Mexico; ^3^ CONAHCYT-Instituto Nacional de Pediatría, Ciudad de México, Mexico; ^4^ Servicio de Oncología, Instituto Nacional de Pediatría, Ciudad de México, Mexico; ^5^ Servicio de Hematología, Instituto Nacional de Pediatría, Ciudad de México, Mexico; ^6^ Departamento de Metodología de la Investigación, Instituto Nacional de Pediatría, Ciudad de México, Mexico

**Keywords:** pre-B acute lymphoblastic leukemia, Mexicans, children, overweight, obesity, *CRLF2* overexpression, SNPs in *GATA3*

## Abstract

**Introduction:**

*CRLF2* abnormalities are prevalent in Hispanics from the U.S. and Mexican children with pre-B acute lymphoblastic leukemia (ALL). This trait is associated with unfavorable prognosis. Furthermore, SNPs rs3824662 and rs3781093 in *GATA3* have been associated with an increased risk of pre-B ALL. In particular, rs3824662 is associated with *CRLF2*-ALL, with a higher prevalence in Hispanic patients. Additionally, rs3824662 is associated with adipogenesis, since Hispanic patients have a high prevalence of obesity and overweight, it has been suggested that obesity predisposes to *CRLF2-*ALL.

**Methods:**

In this study, we evaluated rs3824662 and rs3781093 as predisposition markers for pre-B ALL in Mexican children using Taqman probes.

**Results:**

Both risk alleles were found to be associated with susceptibility to pre-B ALL, predisposition to *CRLF2*-ALL, overweight status, and overall survival. The risk alleles of both SNPs in Mexican patients were among the most frequent compared with other non-Amerindian populations. SNP rs3824662 and rs3781093 were informative for our patients. Analysis of nutritional status indicated that *GATA3* alleles may impact overweight status.

**Discussion:**

Further studies on the relationship between nutritional status and *GATA3*, as well as analysis of other Amerindian ALL populations, are recommended.

## 1 Introduction

Environmental and genetic factors are considered promoters of childhood acute lymphoblastic leukemia (ALL) (1, 2). Currently, several single nucleotide polymorphisms (SNPs) in numerous genes, such as rs3824662 and rs3781093 in *GATA3* (GATA-binding protein 3), have been associated with the risk of developing this disease; for these SNPs the risk alleles described are A and C respectively (1). In particular, the germline variant rs3824662 has been widely studied in ALL patients from different populations worldwide and has been associated with the high-risk Ph-like subtype, which is part of the precursor B (pre-B) ALL. In this context, the risk homozygote AA at rs3824662 has been associated with poor prognosis in children and adolescents, and few studies have replicated the findings described for rs3781093 (3, 4).

*GATA3* encodes a transcription factor that regulates T-cell development and contributes to determining the identity of hematopoietic cells (1). SNP rs3824662 is located in the transcription enhancer region of *GATA3* and is considered a cis-acting regulatory element that increases its expression. At the same time, *GATA3* overexpression induces the expression of the leukemia oncogene *CRLF2* (cytokine receptor-like factor 2), which is altered in 50% of Ph-like patients. In contrast, rs3781093 C risk allele did not affect *GATA3* transcription (3, 5).

The SNPs in *GATA3* appear to have more influence on ALL susceptibility depending on the ethnic origin of patients; in this regard, Hispanic patients living in the U.S. present a higher frequency than Caucasian, Asian, and African patients (6). Inherited *GATA3* variants are associated with Ph-like childhood ALL and the risk of relapse (2, 6).

Interestingly, Hispanic and Mexican patients with pre-B ALL also present a higher frequency of *CRLF2* lesions, such as *P2RY8::CRLF2* and *IGH::CRLF2* rearrangements or *CRLF2* overexpression, than Caucasian, Asian, and African-American patients (7–9). Based on this, Mexican patients could present a higher frequency of risk alleles in *GATA3*; nevertheless, this has not been determined in pre-B ALL cases or in a healthy population.

An independent study showed that Hispanic patients with *CRLF2* rearrangements had higher obesity rates than those without *CRLF2* lesions. This finding suggests that obesity and *GATA3* risk alleles may contribute to *CRLF2* altered pre-B ALL (*CRLF2*-ALL) leukemogenesis and maintenance, through obesity-induced phosphatidylinositol 3-kinase (PI3K)/AKT and mTOR signaling (10). However, *GATA3* risk alleles have not yet been determined in overweight patients with *CRLF2-*ALL.

As germline risk variants in *GATA3* co-segregate with specific somatic abnormalities in pre-B ALL (11), the aim of this study was to determine rs3824662 and rs3781093 as disease predisposition markers in Mexican patients. Here, we associated the *GATA3* risk alleles with: a) susceptibility to pre-B ALL; b) the risk of developing pre-B ALL concomitantly with the overexpression of *CRLF2*; c) the nutritional status of pre-B ALL patients; and d) the event-free survival (EFS) and overall survival (OS) of patients.

## 2 Materials and methods

### 2.1 Patients and controls

A total of 130 patients aged <18 years who were diagnosed with pre-B ALL were included in the study (**Supplementary Table 1**). Patients were recruited at the time of diagnosis in the Oncology and Hematology Departments of the National Pediatrics Institute in Mexico City. The diagnosis was established using cytomorphology, immunophenotyping, and molecular biology for the most common gene fusions. Clinical and laboratory data were obtained from clinical records. The control group consisted of 130 unselected, healthy, unrelated adults with no family history of hematological malignancies. The patients and controls were Mexican mestizo residents in Mexico, with parents and grandparents born in Mexico. Patients, parents or legal tutors signed an informed consent form following the guidelines of the Declaration of Helsinki. The Institutional Research and Ethics Committee approved this study (project 076/2019; National Commission of Bioethics registration number: CONBIOETICA-09-CEI-025–20161215).

### 2.2 Biological samples

Saliva was obtained from pre-B ALL patients (Oragene DNA kit, DNA Genotek Inc. Ottawa, ON, Canada), and saliva or peripheral venous blood (EDTA-supplemented tubes) from the controls. Genomic DNA was extracted from saliva and blood samples (prepIT-L2P kit, DNA Genotek Inc. Ottawa, ON, Canada, and the QIAamp DNA Blood kit, QIAGEN, Hilden, Germany).

Bone marrow samples were collected from the patients with pre-B ALL at the time of diagnosis. *CRLF2* expression (mRNA) was evaluated using TaqMan probes and *GATA3* expression was detected in the same manner. Additionally, we determined *P2RY8-CRLF2* rearrangement by RT-PCR, and when possible, samples with high *CRLF2* expression and negative for *P2RY8::CRLF2* were analyzed for *IGH::CRLF2* rearrangement by fluorescence *in situ* hybridization (FISH).

### 2.3 Genotypification of rs3824662 and rs3781093 in *GATA3*

Both SNPs were genotyped by real-time PCR (StepOne Real-Time PCR, Applied Biosystems, Foster City, CA, U.S.) under standard conditions using predesigned TaqMan probes (VIC/FAM dye-labeled fluorescent probes; Applied Biosystems, Foster City, CA, U.S. ID C:27522049_10 and C:25809980_10, respectively). Each experiment included negative and positive controls for each genotype. Amplification was repeated randomly in 10% of the samples, and concordance was observed.

### 2.4 Analysis of *CRLF2* and *GATA3* expression

RNA was extracted from mononuclear cells in bone marrow samples using (RNeasy kit Qiagen, Düsseldorf, Germany) and cDNA was obtained using standard methods (Invitrogen, Waltham, MA, U.S.). The relative gene expressions of *CRLF2*, *GATA3*, and *GUSβ* (endogenous control) were determined in duplicate by real-time RT-PCR (LightCycler 2.0 Instrument; Roche Applied Science, Penzberg, Upper Bavaria, Germany) using TaqMan gene expression probes (**Supplementary Table 2**) from the Universal Probe Library System (Roche Applied Science, Penzberg, Upper Bavaria, Germany). *CRLF2* overexpression was established according to the previously described criteria (12). It should be noted that the cutoff value was aligned with the *IGH-CRLF2* positive patient with lower *CRLF2* expression.

### 2.5 Detection of *CRLF2* rearrangements

*P2RY8::CRLF2* was assessed as previously described (13). *IGH::CRLF2* was evaluated by FISH in interphase nuclei and metaphases using the dual-color break-apart probes LSI *IGH* (Abbott Molecular, Chicago, ILL, U.S.) and *CRLF2* (CytoCell–OGT, Oxford, UK), following the manufacturer’s recommendations.

### 2.6 Nutritional status

Anthropometric assessment included height (cm) and weight (kg), which were measured using standard methods. Nutritional status was assessed using the following indicators from the WHO Anthro platform: weight/height (W/H), height/age (H/A), and body mass index (BMI). The classification was made according to the values established in the Official Mexican Standard NOM-008-SSA2-1993 (Control of Nutrition, Growth, and Development of Children and Adolescents). The cut-off points according to the Z score were: 1) Height for age (high +1.99 to +3; normal ≥ -1 to <+1; low -1 and lower). 2) Weight for height (obesity/overweight +1 to +3; normal ≥ -1 a <+1; malnutrition -1 and lower). 3) BMI for age (obesity/overweight ≥ +1; normal ≥ -1 a <+1; malnutrition ≥-1 and lower).

### 2.7 Statistical analysis

Both SNPs were analyzed for deviation from Hardy-Weinberg equilibrium (DeFinetti software (https://ihg.gsf.de/cgi-bin/hw/hwa2.pl), and the genotype and allelic frequencies were calculated for controls and patients. A two-tailed Fisher’s exact test was used to compare differences between groups (GraphPad Software, Inc. La Jolla, CA, U.S.). Odds ratios (OR) with 95% confidence intervals were calculated to estimate the risk of developing childhood pre-B ALL or pre-B ALL with higher *CRLF2* expression in the presence of risk alleles and genotypes (DeFinetti Software). The association between each SNP and susceptibility to pre-B ALL was determined using p-values. *CRLF2* and *GATA3* expression levels were associated with different genotypes using the Kruskal–Wallis nonparametric test (IBM SPSS 29.0, Inc., Chicago, IL, USA). Nutritional status and *GATA3* genotype associations were calculated using the chi-squared test. EFS and OS were calculated for patients with different genotypes using the Kaplan–Meier method and Cox regression analysis for hazard ratios (IBM SPSS 29.0). For all comparisons, statistical significance was set at p ≤ 0.05.

## 3 Results

Clinical and laboratory data such as gender, age, white blood cell count, leukemic infiltration site, and presence of gene fusion of the 130 patients included in this study are presented in **Supplementary Table 1**.

### 3.1 Germline variants in *GATA3* and pre-B ALL susceptibility

A total of 130 patients and 130 controls were studied for pre-B ALL susceptibility. For rs3824662 and rs3781093, the genotypic frequencies of the risk homozygotes AA and CC were higher in the patients than in the controls (38.5% vs. 16.2% and 38% vs. 16.9%, p ≤ 0.0001 and p=0.0002, respectively). Similar results were observed for the frequencies of the two risk alleles A and C (0.46 vs. 0.62, p=0.0003 and 0.46 vs. 0.63, p=0.0002, respectively). In contrast, the genotypic frequencies of the non-risk homozygotes CC and TT were higher in controls than in patients (24.6% vs. 14.6%, p=0.0602 and 24.6% vs. 12.4%, p=0.0159). Similar results were obtained for the frequencies of non-risk alleles C and T (0.54 vs 0.38 and 0.54 vs 0.37, respectively) (**Table 1**). OR analysis revealed that risk alleles A and C confer susceptibility to the development of childhood pre-B ALL in our population (OR=1.92, p=0.0002 and OR=1.96, p=0.0002, respectively), and the risk was increased in risk homozygotes (AA OR=4.01, p=0.0004 and CC OR=4.45, p=0.0002, respectively) (**Table 1**).

**Table 1:** Genotypic and allelic frequencies of rs3824662 and rs3781093 in *GATA3*.

Controls vs. Pre-B ALL patients							
SNP rs3824662				SNP rs3781093			
Genotypes and alleles	Controls N=130 (%)	Pre-B ALL patients N=130 (%)	p	Genotypes and alleles	Controls N=130 (%)	Pre-B ALL patients N=129 (%)	p
**CC**	32 (24.6)	19 (14.6)	0.0602	**TT**	32 (24.6)	16 (12.4)	**0.0159**
**AC**	77 (59.2)	61 (46.9)	0.0621	**TC**	76 (58.5)	64 (49.6)	0.1711
**AA**	21 (16.2)	50 (38.5)	**<0.0001**	**CC**	22 (16.9)	49 (38)	**0.0002**
**Alleles** **C**	0.54	0.38	**0.0003**	**Alleles** **T**	0.54	0.37	**0.0002**
**A (risk)**	0.46	0.62		**C (risk)**	0.46	0.63	
Pre-B ALL patients without *CRLF2* overexpression and with *CRLF2* overexpression							
SNP rs3824662				SNP rs3781093			
Genotypes and alleles	No *CRLF2*-OE N=63 (%)	*CRLF2*-OE N=52 (%)	p	Genotypes and alleles	No *CRLF2*-OE N=63 (%)	*CRLF2*-OE N=51 (%)	p
**CC**	13 (20.6)	5 (9.6)	0.1270	**TT**	11 (17.5)	4 (7.8)	0.1681
**AC**	32 (50.8)	19 (36.5)	0.1364	**TC**	33 (52.4)	21 (41.2)	0.2616
**AA**	18 (28.6)	28 (53.8)	**0.0075**	**CC**	19 (30.1)	26 (51)	**0.0337**
**Alleles** **C**	0.46	0.28	**0.0062**	**Alleles** **T**	0.44	0.28	**0.0194**
**A (risk)**	0.54	0.72	**C (risk)**	0.56	0.72
Association with Pre-B ALL susceptibility							
SNP rs3824662				SNP rs3781093			
	OR	OR 95% CI	p		OR	OR 95% CI	p
**C vs A**	1.9269	1.3585-2.7332	**0.0002**	**T vs C**	1.9688	1.3860-2.7965	**0.0002**
**CC vs CA**	1.3342	0.6899-2.5805	0.3915	**TT vs TC**	1.6842	0.8481-3.3447	0.1364
**CA vs AA**	3.0055	1.6323-5.5338	**0.0004**	**TC vs CC**	2.6449	1.4472-4.8337	**0.0016**
**CC vs AA**	4.0100	1.8699-8.5994	**0.0004**	**TT vs CC**	4.4545	2.0358-9.7472	**0.0002**
Association with susceptibility to Pre-B ALL with *CRLF2* overexpression							
SNP rs3824662				SNP rs3781093			
	OR	95% CI	p		OR	95% CI	p
**C vs A**	2.2059	1.2682-3.8370	**0.0051**	**T vs C**	1.9500	1.1184-3.3999	**0.0185**
**CC vs CA**	1.5438	0.4756-5.0105	0.4698	**TT vs TC**	1.7500	0.4922-6.2219	0.3872
**CA vs AA**	2.6199	1.1536-5.9501	**0.0214**	**TC vs CC**	2.1504	0.9607-4.8135	0.0626
**CC vs AA**	4.0444	1.2313-13.2852	**0.0213**	**TT vs CC**	3.7632	1.0377-13.6468	**0.0438**

N, number of analyzed patients; without *CRLF2* overexpression, No *CRLF2*-OE; with *CRLF2* overexpression, *CRLF2*-OE; confidence interval, CI; OR, odd ratio. significant p-values are in bold type.

### 3.2 Germline variants in *GATA3* and pre-B ALL with *CRLF2* overexpression

Sixty-three patients without *CRLF2* overexpression (No *CRLF2*-OE) and 51/52 patients with *CRLF2* overexpression (*CRLF2*-OE) were analyzed (**Table 1**). Both risk homozygotes AA and CC were associated with pre-B ALL with *CRLF2*-OE (53.8% vs. 28.6%, p=0.0075 and 51% vs. 30.1%, p=0.0337, respectively). Risk alleles A and C were also more frequent in *CRLF2*-OE patients (**Table 1**). In comparison, in patients without overexpression (No *CRLF2*-OE), there was a trend towards a higher frequency of the non-risk alleles (C and T) and the non-risk homozygote genotypes (CC and TT) (**Table 1**). OR analysis revealed an association between the risk alleles of rs3824662 and rs3781093, and a predisposition to develop pre-B ALL with *CRLF2*-OE (**Table 1**). The genotypic and allelic frequencies of risk homozygotes (AA and CC) and risk alleles (A and C) for both SNPs were higher in patients with *CRLF2*-OE (53.8% and 51%/0.72 and 0.72, respectively), followed by the total pre-B ALL patients (38.5% and 38.0%/0.62 and 0.63) and the group of patients without *CRLF2*-OE (28.6% and 30.1%/0.54 and 0.56).

### 3.3 rs3824662 and rs3781093 genotypes and *CRLF2* and *GATA3* expression

**Figure 1:**
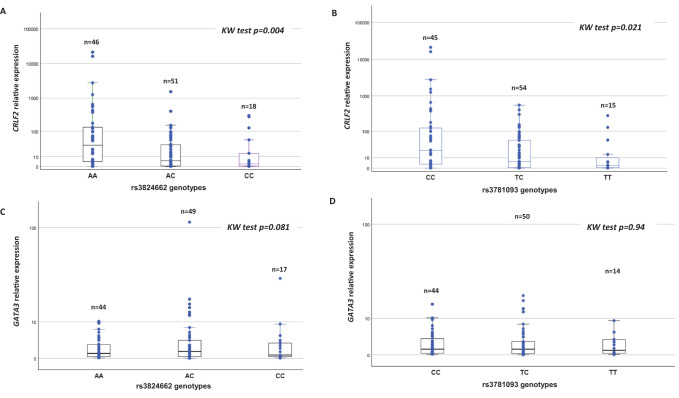
Association of rs3824662 and rs3781093 genotypes with *CRLF2* and *GATA3* expression.

Based on the availability of patient samples, it was possible to analyze 115 of 130 patients for *CRLF2* expression and genotype of rs3824662. The highest levels of *CRLF2* expression were observed in patients with the AA genotype for rs3824662 (p=0.004) compared to patients with AC and CC genotypes (**Figure 1A**). For rs3781093 and *CRLF2* expression 114 patients were successfully studied. Similarly, patients with higher *CRLF2* expression presented with the CC genotype (p=0.021) compared to patients with the TC and TT genotypes (**Figure 1B**). *GATA3* expression was analyzed in 110 patients for rs3824662 and 108 patients for rs3781093; the expression levels detected among genotypes for both SNPs were heterogeneous. No associations were found between the genotypes of either SNP or *GATA3* expression (**Figures 1C, D**), since expression levels of the three genotypes were heterogeneous. In one patient heterozygous for both SNPs, overexpression of *GATA3* was ten orders of magnitude higher than in the other patients. The biological cause of this overexpression has not been investigated at this time, this sample did not show an increase in *CRLF2* expression.

### 3.4 rs3824662 and rs3781093 genotypes and nutritional status

We determined the nutritional status at diagnosis of 66 patients genotyped for rs3824662 and 64 for rs3781093 (**Table 2**). The most frequent nutritional status was normal (41 for both SNPs), followed by malnutrition, 16 and 15, and obese/overweight, 9 and 8 for rs3824662 and rs3781093 respectively. The proportion of risk homozygous genotypes AA and CC (**Table 2**) was higher in obese/overweight patients than in those with malnutrition and adequate nutritional status (p=0.004 and p=0.011, respectively).

**Table 2:** Association of rs3824662 and rs3781093 in *GATA3* with nutritional status in Pre-B ALL patients.

SNP rs3824662 (N=66)				SNP rs3781093 (N=64)			
	Obesity/Overweight (%)	Malnutrition (%)	Normal (%)		Obesity/Overweight (%)	Malnutrition (%)	Normal (%)
**AA (risk)**	8 (89)	7 (44)	9 (22)	**CC (risk)**	7 (88)	7 (47)	10 (24)
**CA**	1 (11)	7 (44)	22 (54)	**TC**	1 (12)	7 (47)	22 (54)
**CC**	0	2 (12)	10 (24)	**TT**	0	1 (6)	9 (22)
**Total**	9 (14)	16 (24)	41 (62)	**Total**	8 (13)	15 (23)	41 (64)
**p*=*0.004**				**p*=*0.011**			

significant p-values are in bold type

### 3.5 Survival analysis

**Figure 2:**
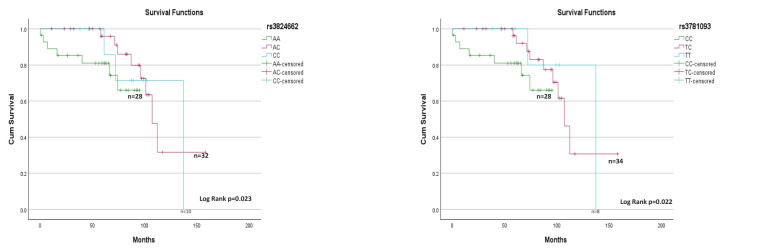
rs3824662 and rs3781093 genotypes and overall survival (n=70).

For survival analysis, patients who discontinued chemotherapy before relapse and patients with a follow-up of <60 months were excluded. The treatment protocols for the evaluated patients were based on the Pediatric Medical Insurance Program (14). Based on this, 70 patients were included in the survival analysis. The risk homozygote genotypes AA and CC were not associated with increased EFS (**Supplementary Figure 1**); however, a positive association (**Figure 2**) with OS was observed for rs3824662 and rs3781093 (p=0.023 and p=0.022, respectively). We looked for associations between patients with risk genotypes and rearrangements in *CRLF2* and survival, but did not observe any difference.

## 4 Discussion

### 4.1 Genotypes and alleles

**Figure 3:**
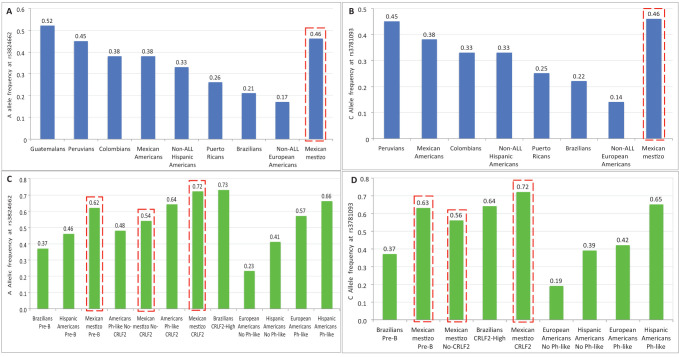
Allele frequencies of the risk alleles at rs3824662 and rs3781093 in different American populations. **(A, B)** correspond to controls and **(C, D)** to pre-B ALL children. Data from Colombians, Peruvians, Mexican Americans and Puerto Ricans were obtained from the 1000 Genomes Project (15). Data from Brazilians, Guatemalans, European Americans, Hispanic Americans, American controls and ALL children were obtained from Perez-Andreu, et. al., and (6) Migita, et. al., (16). (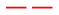) Data from this study.

Regarding the risk alleles A and C of both SNPs in *GATA3*, the frequencies in control populations varied widely throughout the Americas. For rs3824662, the higher frequency was observed in Guatemalans, followed by Peruvians, Colombians, Mexican Americans and Hispanic Americans (0.52, 0.45, 0.38, 0.38 and 0.33, respectively) (6, 15). In populations such as Puerto Ricans, Brazilians, and European Americans, the frequencies were lower (0.26, 0.21, and 0.17, respectively) (**Figure 3A**) (6, 15, 16). The frequency of the A allele in Mexican mestizos from this study (0.46) was close to those observed in Guatemalans and Peruvians, therefore it can be considered among the higher frequencies documented (**Figure 3A**). For rs3781093, the C allele frequencies in controls were higher in Peruvians, Mexican Americans, Colombians, and Hispanics (0.45, 0.38, 0.33, and 0.33, respectively), compared to Puerto Ricans (0.25), Brazilians (0.22), and European Americans (0.14) (6, 15, 16), but none of these were higher than that found in Mexican mestizos (0.46) (**Figure 3B**). It is possible that Brazilian controls have a lower frequency of risk alleles because the SNPs studied are poorly associated with African ancestry, which is predominant in this population (6, 17, 18).

In childhood patients with pre-B ALL, few studies have reported the frequencies of both alleles in Latin Americans, therefore comparisons between populations are difficult. The ascending order of frequency for the A allele at rs3824662 was as follows: Brazilians (0.37), Hispanics (0.46), and Mexican mestizos (0.62) (6, 16) (**Figure 3C**). As expected, the frequency of the C allele in rs3781093 was similar to that found for rs3824662 (16) (**Figure 3D**). Considering the frequencies reported in different childhood pre-B ALL subtypes, the A allele was more frequent in Brazilians with *CRLF2*-high (0.73) than in *CRLF2*-Mexican mestizos (0.72), Ph-like *CRLF2*-Americans (0.64), No-*CRLF2*-Mexican mestizos (0.54), and Ph-like No*-CRLF2*-Americans (0.48) (6, 16) (**Figure 3C**). For rs3781093, the C allele frequency in patients was lower in No-*CRLF2* Mexican mestizos (0.56) than in Brazilians with *CRLF2*-High and *CRLF2*-Mexican mestizos (0.64 and 0.72) (16) (**Figure 3D**). These results show that the risk alleles in rs3824662 and rs3781093 are overrepresented in our population and are associated with the risk of developing pre-B ALL and *CRLF2*-ALL. This suggests that the Amerindian component of Mexicans may be important for the high frequency of this subtype of leukemia in our population.

### 4.2 Survival and genotypes

A positive association was observed only with OS for both the SNPs, this result may be influenced by the sample size, as patients who temporarily discontinued treatment and those with less than 60 months of follow-up were excluded from the analysis. In our setting, it is of utmost importance to implement follow-up measures for patients at risk of discontinuation and nonadherence (19).

### 4.3 Expression assays

To our knowledge, the *CRLF2* OE has only been reported in AA variant carriers (20); in our cohort, the association with OE was found not only for AA but also for CC. It has been observed that rs3824662 upregulates *GATA3* transcription, which alters chromatin accessibility, indicating that *GATA3* potentiates *CRLF2* expression (11). Regarding *GATA3*, no increased expression was found in patients who were homozygous for the risk alleles of the two SNPs analyzed. In contrast to previous studies performed in the HapMap cell lines from different populations and in patient lymphoblasts from the Children’s Oncology Group cohorts (6), in this study, we observed a wide variability in *GATA3* expression among the three genotypes for each SNP. These results are attributed to the different cellular physiological conditions, to undetected genetic abnormalities in *GATA3*, or to the transcriptional or epigenetic regulation present in the leukemic blasts of each patient. In addition, it is important to note that the increased enhancer activity was only reported for the rs3824662 risk allele, while the rs3781093 allele did not appear to have the same effect (21).

### 4.4 Nutritional status

The high rate of obesity in pre-B ALL Hispanic patients living in the U.S. has been considered a predisposing factor for the occurrence of *CRLF2-*ALL (10). It has been suggested that these characteristics may be related to the presence of the risk allele at rs3824662, which may disrupt adipogenesis, metabolism, and/or signaling pathways that contribute to the development of *CRLF2* pre-B ALL (10). However, the authors did not report SNP genotyping data.

In this study, the risk homozygotes of both SNPs (AA and CC) were associated with the overweight status of the patients. To our knowledge, this is the first time that germline variations in *GATA3* have been associated with the nutritional status of the Mexican pre-B ALL patients. Our results are consistent with those observed in Hispanic patients, but they must be considered with caution because: a) the method of determining nutritional status in the previous study was more complete than that used in our patients (fat mass and body fat percentage measured by whole-body dual-energy X-ray absorptiometry vs. anthropometric assessment including height, weight, and BMI); b) the number of patients analyzed for nutritional status in both studies was low; and c) the nutritional characteristics of the two populations studied may be different. The U.S. Hispanic patients have a high prevalence of obesity, whereas Mexican patients who attend our institution are overweight (20%) and malnourished (22%) (22). As expected, this study recruited a higher proportion of malnourished patients, mainly homozygotes or heterozygotes, for the risk alleles. In this context, we suggest that although the association of overweight/obesity with the analyzed risk homozygous genotypes is clear, it should not be excluded that the genotype of *GATA3* variants may partly influence metabolic alterations leading to abnormal nutritional status. It has been noted that there is a link between nutritional changes and GATA3 protein, as it can alter adipogenesis and lead to insulin resistance, and inhibition of GATA3 has been shown to modify impaired adipogenesis and contribute to restoring healthy fat distribution (23). The contribution of nutritional status to ALL development through GATA3 requires further investigation.

## 5 Conclusion

This is the first study to investigate the association between *GATA3* SNPs and predisposition to childhood pre-B ALL and *CRLF2*-ALL in Mexican patients. This confirms the high frequency predicted for the risk alleles in our population and shows that not only the SNP rs3824662, but also rs3781093 have a high penetrance and are effective markers of predisposition for the development of *CRLF2*-ALL, which is common in our patients. It also shows, for the first time, that being overweight, estimated by BMI at the time of patient diagnosis, is associated with the presence of the risk alleles of both polymorphisms. The limitations of this study include the need to refine measures of body mass and fat, to have cohorts of patients with longer follow-up to obtain more reliable survival calculations, and to perform transcriptome sequencing that will allow us to know the alterations associated with ALL Ph-like and the subgroup of patients with an aberrant Jak-Stat pathway. This will enable us to establish more specific associations between groups, allowing us to better understand our population and obtain data that can be extrapolated to populations of Amerindian ancestry.

## Data Availability

The original contributions presented in the study are publicly available. This data can be found here: https://www.ncbi.nlm.nih.gov/clinvar/; SCV006060650 - SCV006060651.
