# Supplementary material for: GATA3 germline variants in childhood pre-B acute lymphoblastic leukemia: association with CRLF2 overexpression and overweight in Mexican patients
Source: Front Oncol. 2025 May 12;15:1533756. doi: 10.3389/fonc.2025.1533756 (PMC12104813; doi:10.3389/fonc.2025.1533756)
Supplement: Supplementary file 1 [file DataSheet1.pdf]

Supplementary Table 1. Clinical and laboratory characteristics of pre-B ALL patients

**Gender**	Male	59
	Female	71
**Age (years)**	1-9	78
	≥10	49
	Unknown	3
**White blood cell count**	<$50X10^9$/L	94
	≥$50X10^9$/L	31
	Unknown	5
**Leukemic infiltration**	Central Nervous System and/or Testicular	25
	Other site	1
**Gene fusions**	*BCR::ABL1*	1
	ETV6::RUNX1	5
	TCF3::PBX1	6

Supplementary Table 2. Taqman gene expression probes (design from ProbeFinder version 2.45 Human)

Gene	Primer Reverse 5´-3´	Primer Forward 5´-3´	Probe number
CRLF2 NM_022148.1	AATTTGGACAGCTTTGGTTTG	AGCGACTGGTCAGAGGTGA	53
GATA3 NM_001002295	CCCTCATTAAGCCCAAGCGA	AGCGACTGGTCAGAGGTGA	71
GUSB NM_000181.3	TCCCCACAGGGAGTGTGTAG	CGCCCTGCCTATCTGTATTC	57
