# Supplementary figures and images for: GATA3 germline variants in childhood pre-B acute lymphoblastic leukemia: association with CRLF2 overexpression and overweight in Mexican patients
Source: Front Oncol. 2025 May 12;15:1533756. doi: 10.3389/fonc.2025.1533756 (PMC12104813; doi:10.3389/fonc.2025.1533756)

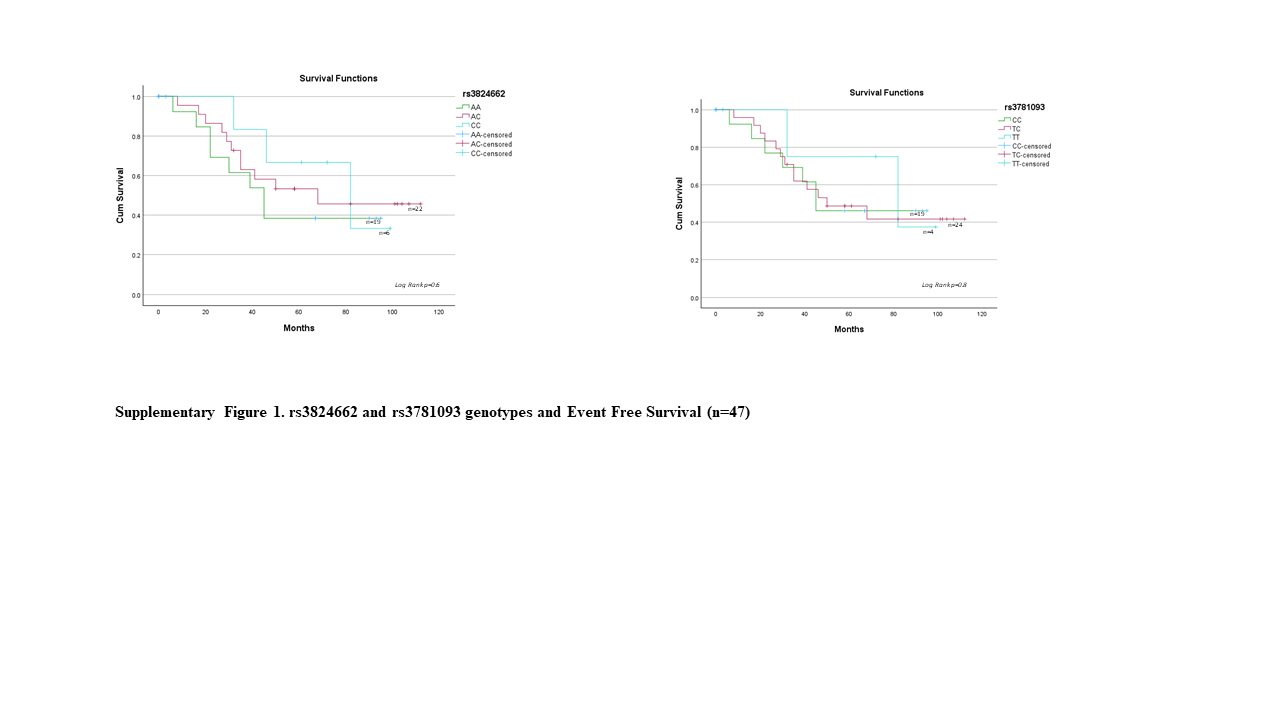

Supplement: Supplementary file 2 [file Image1.tif]
